# Artemisinin ameliorates rheumatoid arthritis through modulation of the IL-17/PI3K/AKT signaling pathway: integrated network pharmacology, bioinformatics, and experimental validation

**DOI:** 10.3389/fimmu.2026.1862104

**Published:** 2026-08-13

**Authors:** Zongyuan Zhou, Le Wang, Yongzuo Li, Yue Yang, Lingyu Li, Xing Qin, Thomas Efferth, Xiaohua Lu, Zhe Qiang

**Affiliations:** 1 Antibiotic Innovation and Resistance Control Key Laboratory of Sichuan Province, National Base for International Science and Technology Cooperation, School of Pharmacy, Chengdu University, Chengdu, China; 2 Affiliated Hospital of Chengdu University, Chengdu University, Chengdu, China; 3 Center for Natural Products Research, Chengdu Institute of Biology, Chinese Academy of Sciences, Chengdu, China; 4 Department of Pharmaceutical Biology, Institute of Pharmaceutical and Biomedical Sciences, Johannes Gutenberg University Mainz, Mainz, Germany; 5 Sichuan-Chongqing Joint Key Laboratory of Innovation of New Drugs of Traditional Chinese Medicine, Chongqing Academy of Chinese Materia Medica, Chongqing University of Chinese Medicine, Chongqing, China

**Keywords:** bioinformatics, IL-17, molecular docking, network pharmacology, PI3K/AKT, rheumatoid arthritis

## Abstract

**Purpose:**

Artemisinin regulates the immune system and is applied in RA treatment. Nevertheless, the effectiveness and potential mechanisms of artemisinin in the treatment of RA remain unclear, especially in fibroblast-like synoviocytes (FLSs). This study aimed to investigate the mechanism of artemisinin in the treatment of rheumatoid arthritis (RA) through an integrated network pharmacology and bioinformatics approach, supported by experimental validation both in MH7A cells and collagen-induced arthritis (CIA) mice.

**Methods:**

SwissTargetPrediction database, TCMSP, TargetNet, SuperPred, and PharmMapper were used to identify the targets of artemisinin. RA-related differentially expressed genes (DEGs) were collected by integrating three GEO datasets. DEGs related to artemisinin were utilized to create protein-protein interaction networks and visualize them with STRING and Cytoscape. GO and KEGG functional enrichment analyses were executed. Molecular docking and molecular dynamics (MD) simulations were carried out to analyze core targets using the AutoDock Vina and Desmond software. Furthermore, CIA mouse and MH7A cells were used to validate the results obtained from network pharmacology.

**Results:**

There were 73 target genes intersecting between the artemisinin targets and the DEGs. PPI network analysis and its topology indicated that AR, EGFR, JAK2, and PTGS2 had the greatest centrality. GO and KEGG enrichment analyses suggested that the candidate targets were significantly associated with the PI3K/AKT signaling pathway. Molecular docking and an in vivo study were used for further investigation. Artemisinin interacted with the cavities of AR, JAK2, EGFR, and PTGS2 with binding energies of –8.65, –8.61, –7.48, and –8.48 kcal/mol, respectively. MD simulations showed that the AR–artemisinin complex maintained persistent interactions and stable energy profiles over 20 ns. The CIA model and MH7A cell studies confirmed that artemisinin exposure inhibited the proliferation and production of IL-1β, IL-6, and IL-8 triggered by IL-17. Furthermore, artemisinin attenuated IL-17-induced AKT phosphorylation levels.

**Conclusion:**

These findings suggest that artemisinin may ameliorate RA partly by suppressing IL-17-induced inflammatory activation and PI3K/AKT signaling. AR, EGFR, JAK2, and PTGS2 were prioritized as candidate hub targets.

## 1 Introduction

Rheumatoid arthritis (RA) is a widespread autoimmune disease, which is defined by systemic inflammation and results in joint hyperplasia, pain, and swelling at the lesion sites (1). It can severely compromise physical function and quality of life. In addition, RA patients are exposed to greater health risks than the general population, for example, serious infections, lung diseases, bone density loss, heart diseases, and even cancer and mortality (2). As a result, RA imposes a considerable economic burden on both individuals and society (3). Within RA medications, disease-modifying antirheumatic drugs (DMARDs) are designed to suppress inflammation and slow down the progression of structural damage in lesions. Additionally, the efficacy of biologic DMARDs is enhanced through combination therapy with methotrexate and conventional synthetic DMARDs (4). However, many patients still fail to achieve sustained remission (4, 5). Notably, the costly treatment of biological DMARDs, the inconvenience of portability, and the serious side effects still affect their clinical utility. The current objective of treating RA is strict remission or minimal disease activity, yet many patients fail to meet this target, meaning that new effective treatments and drugs are urgently needed (6). Therefore, new small-molecule agents with defined anti-inflammatory mechanisms remain needed.

As a first-line antimalarial drug, artemisinin attracted widespread attention after the emergence of chloroquine resistance. As research has progressed, artemisinin has attracted increasing attention for its therapeutic potential in various diseases (7), including RA, although its specific targets and mechanisms remain unclear (8–12). Previous studies have reported the anti-inflammatory and anti-arthritic activities of several artemisinin derivatives (8, 11, 13–15). Artemisinin itself has also been implicated in the regulation of TGF-β1 signaling (16), NLRP3 (17) and NF-κB (18, 19). However, the therapeutic mechanism of artemisinin itself in RA, particularly in RA fibroblast-like synoviocytes (FLSs), remains to be fully elucidated. Here, we investigated artemisinin itself using integrated network pharmacology, RA synovial transcriptomic analysis, molecular docking, and experimental validation in IL-17-stimulated MH7A cells and CIA mice. This integrated strategy allowed us to systematically identify and support the IL-17/PI3K/AKT signaling axis as a potential mechanism underlying the anti-RA effect of artemisinin. Here, molecular docking further suggested potential interactions between artemisinin and selected candidate hub targets, including AR, EGFR, JAK2, and PTGS2. Subsequent experimental validation focused on the IL-17/PI3K/AKT signaling axis in MH7A cells and CIA mice.

## 2 Materials and methods

### 2.1 Methods and materials

Chemical reagents: Artemisinin was obtained from Selleck in Shanghai, China, and dissolved in DMSO. Fetal bovine serum was sourced from ExCell in China. Penicillin/streptomycin and trypsin were purchased from Sanggon (Shanghai, China). Dulbecco’s Modified Eagle Medium (DMEM) was acquired from Hyclone in the USA; Dimethyl sulfoxide (DMSO) was purchased from Beijing Suolaibao Technology (Beijing, China). CCK8 and Protease/Phosphatase Inhibitor Cocktail (MedChemExpress, Shanghai, China). RIPA lysis buffer, BCA Protein Assay Kit, IL-8, IL-6, and IL-1β ELISA kits were sourced from Beyotime (Shanghai, China). Primary antibodies against GAPDH, PTGS2, and AKT and p-AKT were purchased from Proteintech Group, Inc (Hubei, China). ECL luminescence solution was purchased from 4A BIOTECH (Jiangsu, China).

### 2.2 Construction of target genes related to the bioactive compounds

The identification of artemisinin targets was conducted through an integrated approach, leveraging the following prediction platforms: SwissTargetPrediction (http://www.swisstargetprediction.ch/), Traditional Chinese Medicine Systems Pharmacology Database (TCMSP; http://tcmspw.com/tcmsp.php), TargetNet (http://targetnet.scbdd.com/), SuperPred (https://prediction.charite.de/index.php), and PharmMapper (http://www.lilab-ecust.cn/pharmmapper/). Target prediction was specifically constrained to the Homo sapiens species. Subsequently, all retrieved target names underwent standardization via the UniProt database (https://sparql.uniprot.org/).

### 2.3 GEO datasets and data preprocessing

Publicly available gene expression profiles derived from human synovial tissue, encompassing RA patients and healthy donors, were sourced from the Gene Expression Omnibus (GEO) repository hosted by the National Center for Biotechnology Information (NCBI; https://www.ncbi.nlm.nih.gov/geo/). To obtain more effective information from sufficient samples, we integrated the multiple datasets (GSE55235, GSE55457, GSE77298) using the R package “inSilicoMerging”. Further, transcriptomic data underwent batch effect correction, yielding a normalized expression matrix. Using the ‘Limma’ R package, this matrix was analyzed to find DEGs between RA synovial tissue and healthy donors, applying thresholds of |log_2_FC| > 0.585 and p < 0.05. Common targets intersecting with these DEGs were visualized through Venn diagram analysis.

### 2.4 Analysis of GO and KEGG enrichment

To elucidate signaling pathways and biological processes potentially modulated by artemisinin, we performed Gene Ontology (GO) and Kyoto Encyclopedia of Genes and Genomes (KEGG) enrichment analyses on the intersecting gene set derived from artemisinin targets and synovial tissue DEGs. We employed the KEGG REST API (https://www.kegg.jp/kegg/rest/keggapi.html) to retrieve pathway-specific gene annotations, constructing a custom background reference set for KEGG functional enrichment analysis of our gene set. Enrichment analysis was then performed using the R package clusterProfiler (version 3.14.3) to obtain the results of gene set enrichment, and p < 0.05 was considered statistically significant. GO annotations of genes were obtained from the R package org.Hs.eg.db (version 3.1.0) to map genes. We conducted gene set enrichment analysis using the clusterProfiler R package (v3.14.3), with statistical significance defined as p < 0.05. The most enriched GO terms and KEGG pathways were visualized as bar and bubble plots, respectively.

### 2.5 The construction of protein-protein interaction networks

Core regulatory targets were identified through STRING-based PPI analysis (v11.5; https://string-db.org/) of artemisinin-DEG intersection targets under stringent parameters: Homo sapiens restriction, interaction score ≥0.7. Cytoscape 3.8.0, was used to reconstruct the network, in which CytoNCA computed three centrality metrics: betweenness (BC), closeness (CC), and degree (DC). Nodes exceeding median values for all three parameters were defined as key targets and used to generate a refined PPI subnetwork. Hub targets were defined as overlapping nodes ranked highly by degree, betweenness, and closeness centrality; the final four targets were selected based on their shared topological prominence and biological relevance to RA/inflammation.

### 2.6 Molecular docking

As a computational approach fundamental to drug discovery, molecular docking was employed to predict the binding conformations and affinities of artemisinin at protein active sites. High-degree targets in the core PPI network were considered topologically important hub targets. Therefore, AR, JAK2, EGFR, and PTGS2, which showed high degree values in the core PPI network, were prioritized for subsequent docking analysis. Their crystallographic structures were acquired from the RCSB Protein Data Bank (http://www.rcsb.org) using the accession codes AR (2PIV), JAK2 (3TJD), EGFR (5FED), and PTGS2 (5IKQ). The initial two-dimensional (2D) chemical structure of artemisinin was retrieved from the PubChem database (https://pubchem.ncbi.nlm.nih.gov/). Molecular docking was carried out using AutoDock Tools (version 1.5.7) in conjunction with AutoDock Vina (v1.2.0). Preprocessing of the protein structures involved the removal of water molecules and original ligands, addition of hydrogen atoms, and assignment of charges, followed by export in PDBQT format using AutoDock Tools. The 2D structure of artemisinin was converted into the three-dimensional PDB format using Chem3D, followed by hydrogenation, energy minimization, torsion tree optimization, and export as a PDBQT file.

The docking grid boxes were defined to cover the original ligand-binding pockets or predicted active pockets of the target proteins. The exhaustiveness value was set to 8, and 50 binding conformations were generated for each protein–ligand pair. The docking poses were ranked according to their predicted binding affinity scores, and the conformation with the lowest binding energy was selected for further interaction analysis and visualization using PyMOL 2.5. To validate the reliability of the docking protocol, the co-crystallized ligands were removed from their corresponding protein structures and redocked into the original binding pockets under the same docking parameters. The docking procedure was considered reliable when the redocked poses were comparable to the original crystallographic conformations.

### 2.7 Molecular dynamics

Molecular dynamics (MD) simulations were carried out using the Desmond package, a Linux-based platform featuring a visual interface for MD calculations. The XP docking poses were selected as the initial structures for subsequent MD analysis. To construct the simulation system, an explicit solvent model was introduced, and the SPC water model was chosen. An orthorhombic simulation box was generated, with the box dimensions minimized using the buffer method to reduce computational cost. To ensure overall charge neutrality of the system, appropriate counterions (Na^+^ or Cl^-^) were added. The OPLS4 force field was applied for system parameterization. The prepared system was saved in CMS format and then submitted for MD simulation. The production run was performed for 20 ns under the NPT ensemble, with the temperature maintained at 300 K and the pressure set to 1.01 bar. The system energy parameter was defined as 1.2. Before the formal simulation, the entire system was subjected to a relaxation procedure. The final simulation trajectories and related outputs were stored in CMS format.

### 2.8 Cell culture

The fibroblast-like synoviocytes (FLSs)-transformed cell line MH7A (bnbio, Beijing, China) was used to carry out all experiments *in vitro*. The MH7A cell line was maintained under standard culture conditions (37 °C, 5% CO_2_). The culture medium consisted of high-glucose DMEM, enriched with 10% FBS and a 1% antibiotic-antimycotic solution of penicillin-streptomycin. For subculturing, cells were washed with PBS, detached using 0.25% trypsin-EDTA, and seeded at appropriate densities in culture plates.

### 2.9 Cell viability assay

Following 24-hour adherence in 96-well plates (2.5 × 10^4^ cells/well), MH7A cultures were exposed to artemisinin for 24 h. Cellular viability was then quantified via CCK-8 assay: reagent introduction, 60 min incubation, spectrophotometric quantification at λ = 450 nm using a microplate reader (Thermo Scientific).

### 2.10 CIA modeling and artemisinin treatment

Male DBA/1 strain mice were supplied by Dashuo Biotechnology (Chengdu, China) and reared in an SPF facility: 12/12-hour light/dark cycles, 25 ± 2 °C ambient temperature, ad libitum access to standardized rodent chow and autoclaved water. Following seven-day environmental acclimatization, all experimental procedures received ethical approval (NO: CAC20250110) from the Institutional Animal Care and Use Committee at Chongqing Academy of Chinese Materia Medica.

Mice were randomly assigned to four groups (n = 6 per group). To establish the CIA, 8-week-old DBA/1 male mice received an intradermal immunization at the tail base with 100 μL of bovine type II collagen solution (2 mg/mL in 0.1 M acetic acid) emulsified with an equal volume of Complete Freund’s Adjuvant on day 0, followed by a 50 μL booster injection on day 21. Beginning 24 h after the booster immunization (day 22), artemisinin dissolved in sterile corn oil was administered intraperitoneally at 15 or 30 mg/kg every 48 h until day 45. The 30 mg/kg dose was selected based on previous experimental reports and preliminary tolerability considerations, whereas the 15 mg/kg dose was included to evaluate the lower-dose therapeutic response. Disease progression was monitored through tri-weekly clinical scoring (0–4 scale per paw). The hind paw joints were examined using micro-computed tomography (micro-CT; QuantumGX, PerkinElmer, USA) to evaluate structural alterations and joint damage. Clinical scoring, paw thickness measurement, micro-CT evaluation, and histological assessment were performed by investigators blinded to group allocation.

### 2.11 Histological examination

Following euthanasia, the hind paws were excised and fixed in 4% paraformaldehyde to preserve joint morphology. The tibiotarsal joint specimens were subsequently decalcified with EDTA-based decalcifying solution, followed by routine dehydration, clearing, and paraffin embedding. Serial paraffin sections were prepared from the joint region and subjected to hematoxylin and eosin (HE) staining. Histological changes were examined using light microscopy, with particular attention to synovial thickening, inflammatory cell infiltration, cartilage erosion, and destruction of the joint architecture.

### 2.12 Western blotting analysis

Cells were lysed in RIPA buffer containing a protease/phosphatase inhibitor cocktail, and the extracts were centrifuged at 16,000 × g for 20 min at 4 °C to remove insoluble material. The protein concentration of the supernatant was quantified using a BCA assay with bovine serum albumin as the standard, and absorbance was recorded on a Varioskan LUX reader (Thermo Fisher Scientific). Equal protein aliquots (20 μg) were denatured in loading buffer, then resolved on 10% gradient Tris-glycine precast gels in running buffer. Proteins were electrotransferred onto nitrocellulose blotting membranes (Millipore) using a transfer buffer. The membranes were then incubated in TBST containing 5% BSA for 1 h at room temperature to prevent nonspecific binding. Subsequently, primary antibodies were prepared in blocking buffer with the addition of 0.1% Tween-20 and subjected to overnight incubation at 4 °C. Following three 10 min TBST washes, membranes were probed with species-matched secondary antibodies for 1 h at room temperature, protected from light. Blots were imaged using ECL luminescence solution.

### 2.13 ELISA assay

Cell supernatants were harvested from stimulated MH7A cultures (24 h post-treatment) following centrifugation at 1,500 × g for 10 min at 4 °C, supplemented with protease inhibitor cocktail, aliquoted into low-protein-binding tubes, flash-frozen in liquid nitrogen, and stored at -80 °C. As for mouse serum, terminal blood collection via submandibular puncture delivered blood into serum separator tubes. After 30 min of clotting at room temperature, samples were centrifuged at 2,500 × g (15 min, 4 °C) with subsequent supernatant collection as serum. The quantification of IL-1β, IL-6, and IL-8 levels was performed according to the protocols of commercially available ELISA kits.

### 2.14 Data analysis

Results are presented as the mean ± standard deviation (SD). Statistical analyses were performed using GraphPad Prism. Before applying one-way ANOVA, the normality of data distribution and homogeneity of variance were evaluated using the Shapiro–Wilk test and Brown–Forsythe test, respectively. For datasets satisfying these assumptions, multiple-group comparisons were performed using one-way ANOVA followed by Tukey’s multiple comparisons test. A value of p < 0.05 was considered statistically significant.

## 3 Results

### 3.1 The integration of GEO datasets and the removal of batch effects

**Figure 1:**
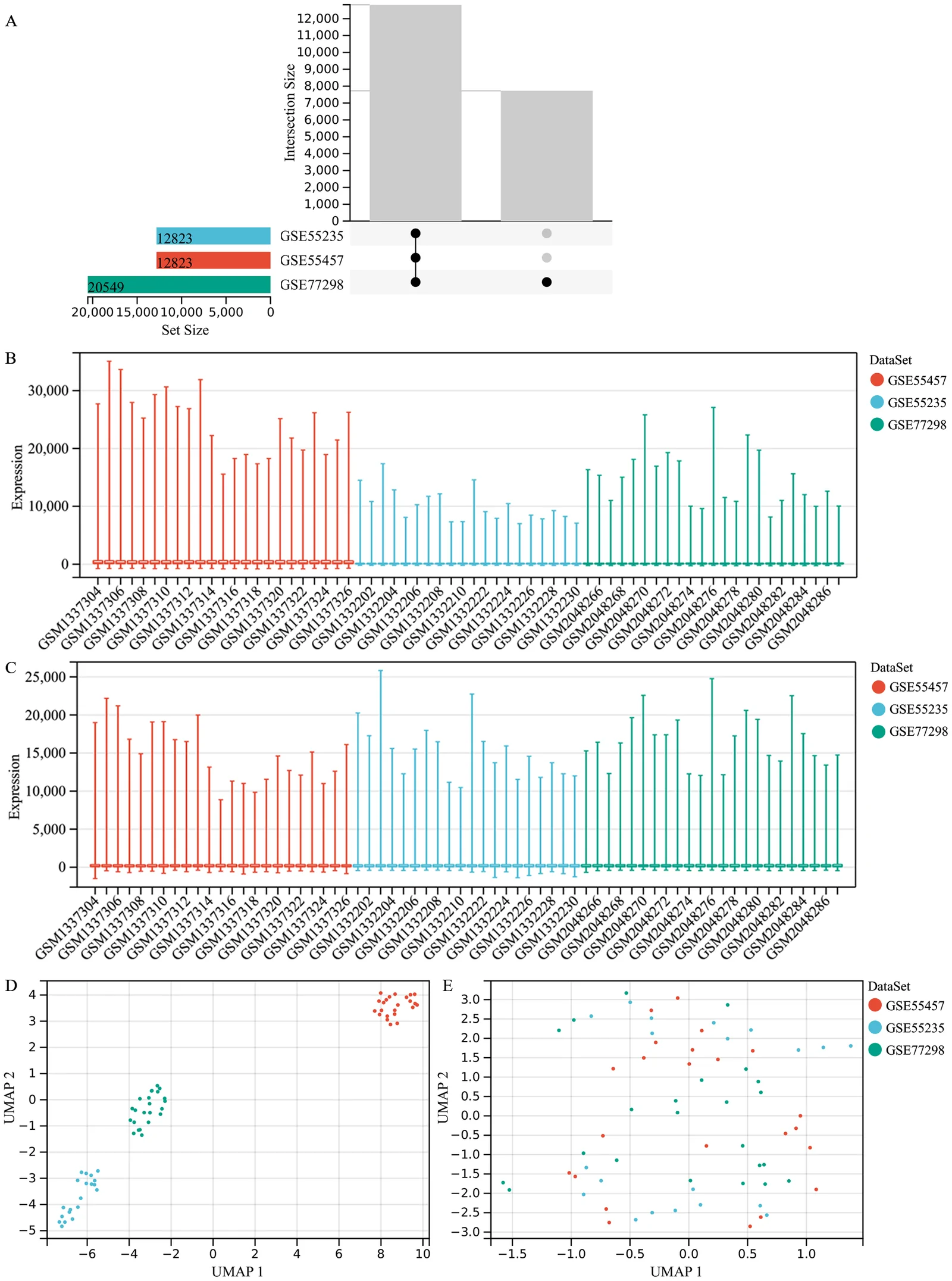
Integration of multiple datasets and removal of batch effects. **(A)** Gene counts across the three datasets. **(B)** Number of expressed genes per sample before integration. **(C)** Number of expressed genes per sample after integration. **(D)** UMAP visualization of sample distribution from the three datasets before batch effect correction. **(E)** UMAP visualization of sample distribution from the three datasets following batch effect removal.

We integrated the multi-datasets based on the GPL570 platform (GSE55235, GSE55457, GSE77298). Osteoarthritis samples were excluded to focus on RA-specific synovial inflammation and avoid degenerative confounding (**Supplementary Table 1**). First, the three datasets were merged, yielding 12,832 overlapping gene expression profiles. After batch-effect correction, a normalized expression matrix was obtained (**Figure 1A**). Further, the new expression matrix was obtained after performing the removal of batch effects. Before removing the batch effect, the samples distributed in the three datasets are widely different (**Figure 1B**), suggesting a batch effect. Post-batch correction, dataset distributions demonstrate visual harmonization, with median values exhibiting linear alignment in comparative boxplots (**Figure 1C**). Meanwhile, UMAP visualization revealed initial dataset segregation before batch correction (**Figure 1D**), whereas post-normalization samples exhibited heterogeneous intermixing across all three datasets (**Figure 1E**).

### 3.2 RA-related DEGs and the target screening of artemisinin

**Figure 2:**
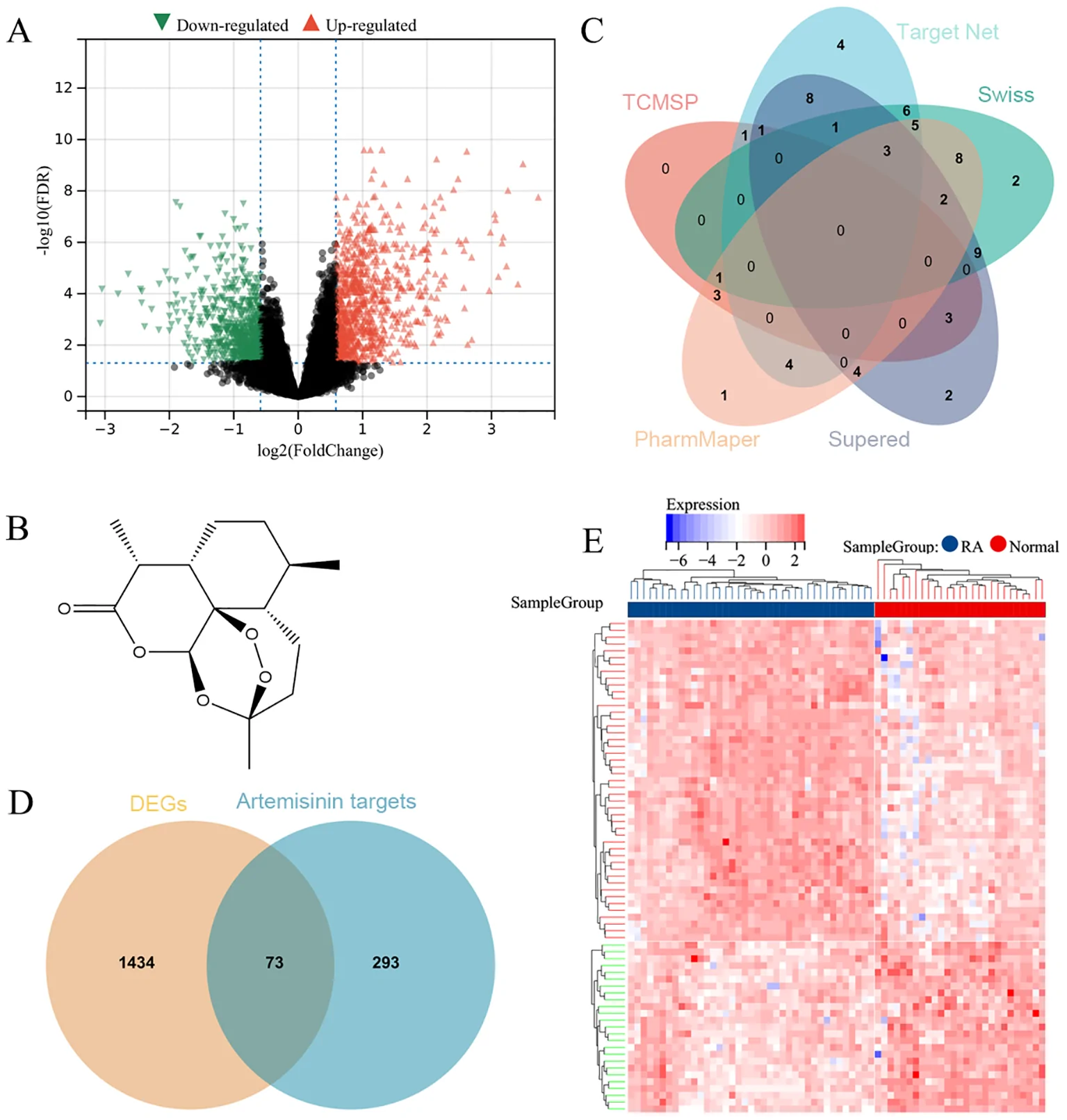
Potential targets of artemisinin in RA treatment. **(A)** Volcano plot displaying differentially expressed genes (DEGs) after batch effect correction. **(B)** The structure of artemisinin. **(C)** Venn diagram showing the number of artemisinin targets identified across different datasets. **(D)** Venn diagram illustrating the intersection between RA differentially expressed genes and artemisinin targets. **(E)** Heatmap of RA-related genes potentially modulated by artemisinin.

Post-integration batch correction yielded a harmonized expression matrix, revealing 844 significantly upregulated and 663 downregulated genes (thresholds: |log_2_FC| > 0.585, false discovery rate (FDR) < 0.05; **Figure 2A**, **Supplementary Table 2**). Artemisinin’s canonical 2D structure (PubChem CID: 68827) was acquired (**Figure 2B**). Multi-source target prediction identified candidate genes through: SwissTarget (n = 96), TCMSP (n = 15), TargetNet (n = 85), SuperPred (n = 147) PharmMapper (n = 102) (**Supplementary Table 3**). Consolidation of 366 non-redundant targets was visualized via Venn analysis (**Figure 2C**). Intersection with RA synovial transcriptomic signatures resolved 73 high-probability targets (**Figure 2D**). Subsequent differential expression analysis demonstrated significant dysregulation of these candidates in RA versus healthy cohorts, evidenced by a hierarchical clustering heatmap (**Figure 2E**).

### 3.3 GO and pathway enrichment analysis

**Figure 3:**
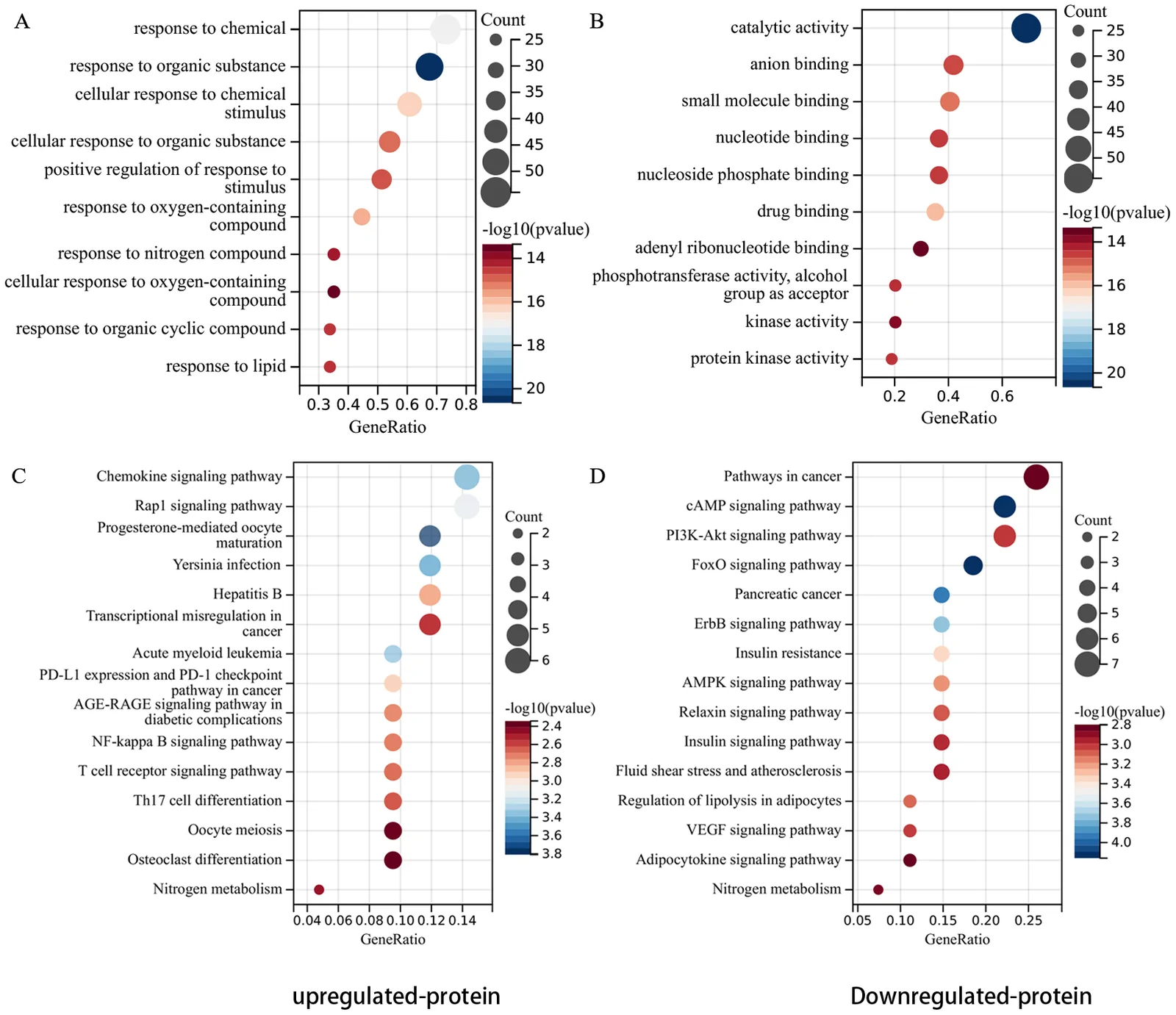
Enrichment analyses of GO and KEGG for genes associated with artemisinin treatment in RA. **(A)** Top ten significantly enriched biological processes. **(B)** Top ten significantly enriched molecular functions. **(C)** KEGG pathway enrichment of up-regulated genes potentially targeted by artemisinin in RA. **(D)** KEGG pathway enrichment of down-regulated genes potentially targeted by artemisinin in RA.

Functional annotation of artemisinin’s putative RA targets was conducted through Gene Ontology (GO) and pathway enrichment analyses. The top ten significantly enriched terms across Gene Ontology categories, including biological processes, molecular functions and cellular components, were visualized through bubble plots (**Figures 3A, B**, **Supplementary Figure 1**). Bubble chart analysis indicated artemisinin’s potential therapeutic mechanisms in RA through modulation of catalytic activity and intermolecular binding functions (**Figure 3B**). To systematically evaluate pathway-level regulation, KEGG enrichment analysis was conducted separately for up- and down-regulated proteins based on target expression changes. The top 10 significantly enriched pathways (FDR < 0.05) for each directional protein set are visualized in **Figure 3C** (up-regulated) and **Figure 3D** (down-regulated). The enrichment results revealed distinct pathway profiles between the upregulated and downregulated target sets, suggesting that artemisinin may exert complex regulatory effects in RA. In detail, artemisinin mostly regulates immune system-related pathways enriched by up-regulated targets including yersinia infection, acute myeloid leukemia, chemokine signaling pathway, PD-L1 expression and PD-1 checkpoint pathway in cancer, T cell receptor signaling pathway and Th17 cell differentiation. As for down-regulated targets, they were mainly enriched in cancer pathway, FOXO signaling pathway, cAMP signaling pathway, ERBB signaling pathway, AMPK signaling pathway, VEGF signaling pathway, PI3K/AKT signaling pathway and so on.

### 3.4 Protein-protein interaction network and hub target identification

**Figure 4:**
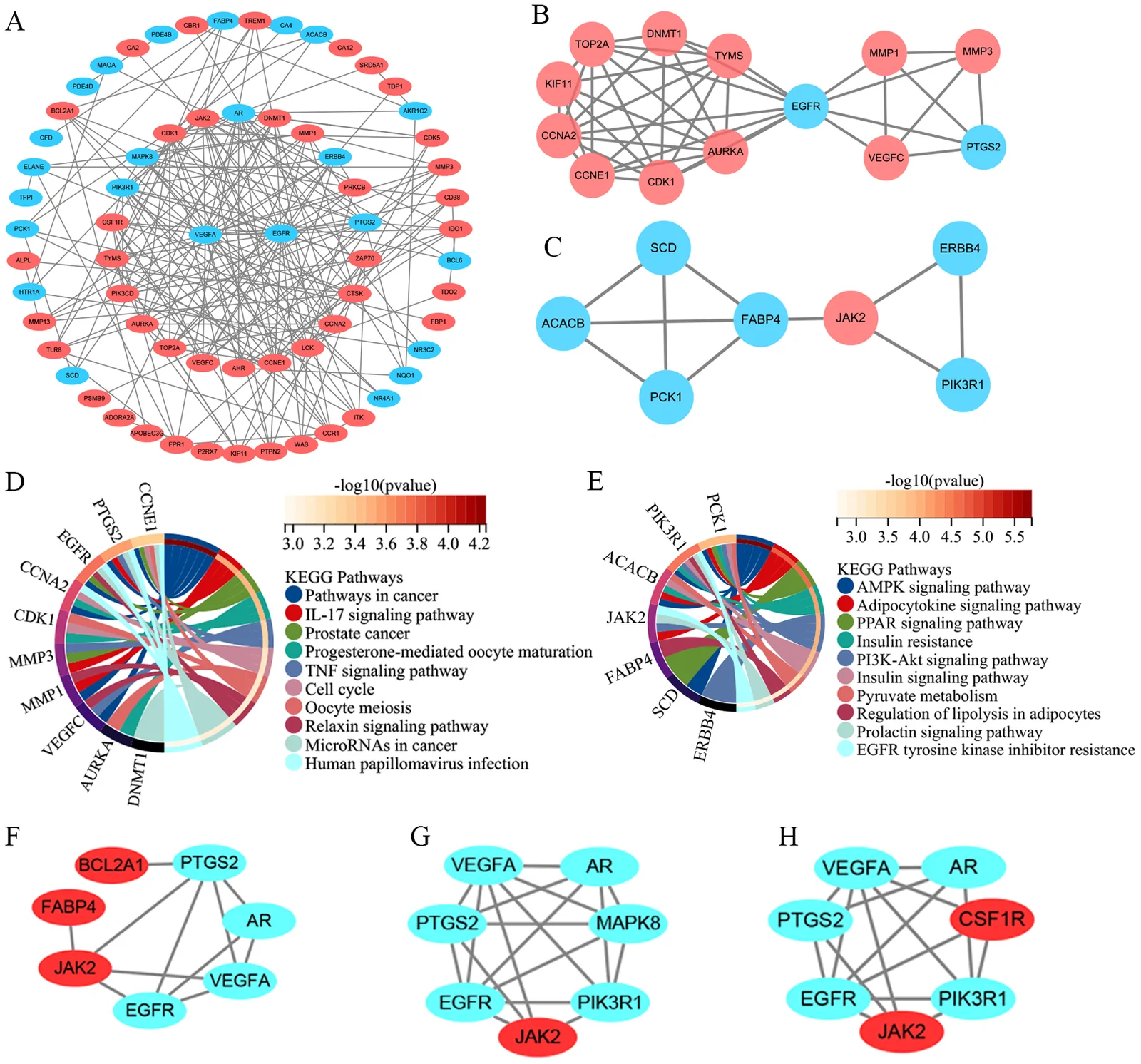
PPI network construction and candidate hub target prioritization. **(A)** PPI network of artemisinin-related RA targets constructed using STRING with an interaction score ≥ 0.7 and visualized in Cytoscape. **(B, C)** Key modules identified by MCODE analysis. **(D, E)** KEGG enrichment analysis of genes in the selected modules. **(F–H)** Hub target screening based on betweenness, closeness, and degree centrality using CytoNCA. AR, EGFR, JAK2, and PTGS2 were prioritized as candidate hub targets based on overlapping topological parameters.

To resolve core therapeutic targets of artemisinin in rheumatoid arthritis, based on interaction data predicted by the STRING database (v11.5), a protein interactome was built and subsequently visualized for graphical representation using Cytoscape (v3.9.1). The resulting network comprised 67 nodes and 424 edges following exclusion of one disconnected target (**Figure 4A**). The identification of coregulatory modules is important for understanding the role of artemisinin, so the network was analyzed in modules using Cytoscape’s MCODE portal. As a result, two modules were carefully chosen based on the mentioned criteria (**Figures 4B, C**). KEGG pathway analysis was then performed for these modules. Module 1 demonstrated significant enrichment in cancer-associated pathways, IL-17 signaling cascade, TNF signaling axis, and cell cycle regulation (**Figure 4D**). Conversely, Module 2 revealed pronounced enrichment in AMPK signaling, adipocytokine signaling, PI3K/AKT signaling cascade, PPAR signaling and EGFR tyrosine kinase inhibitor resistance (**Figure 4E**). After MCODE clustering, topological analysis employing CytoNCA (v0.1) quantified node centrality metrics - betweenness, closeness, and degree centrality across the PPI network (**Figures 4F–H**). Based on the integrated topological analysis, AR, EGFR, JAK2, and PTGS2 were prioritized as candidate hub targets for subsequent in silico validation.

### 3.5 Molecular docking analysis of candidate hub targets

**Figure 5:**
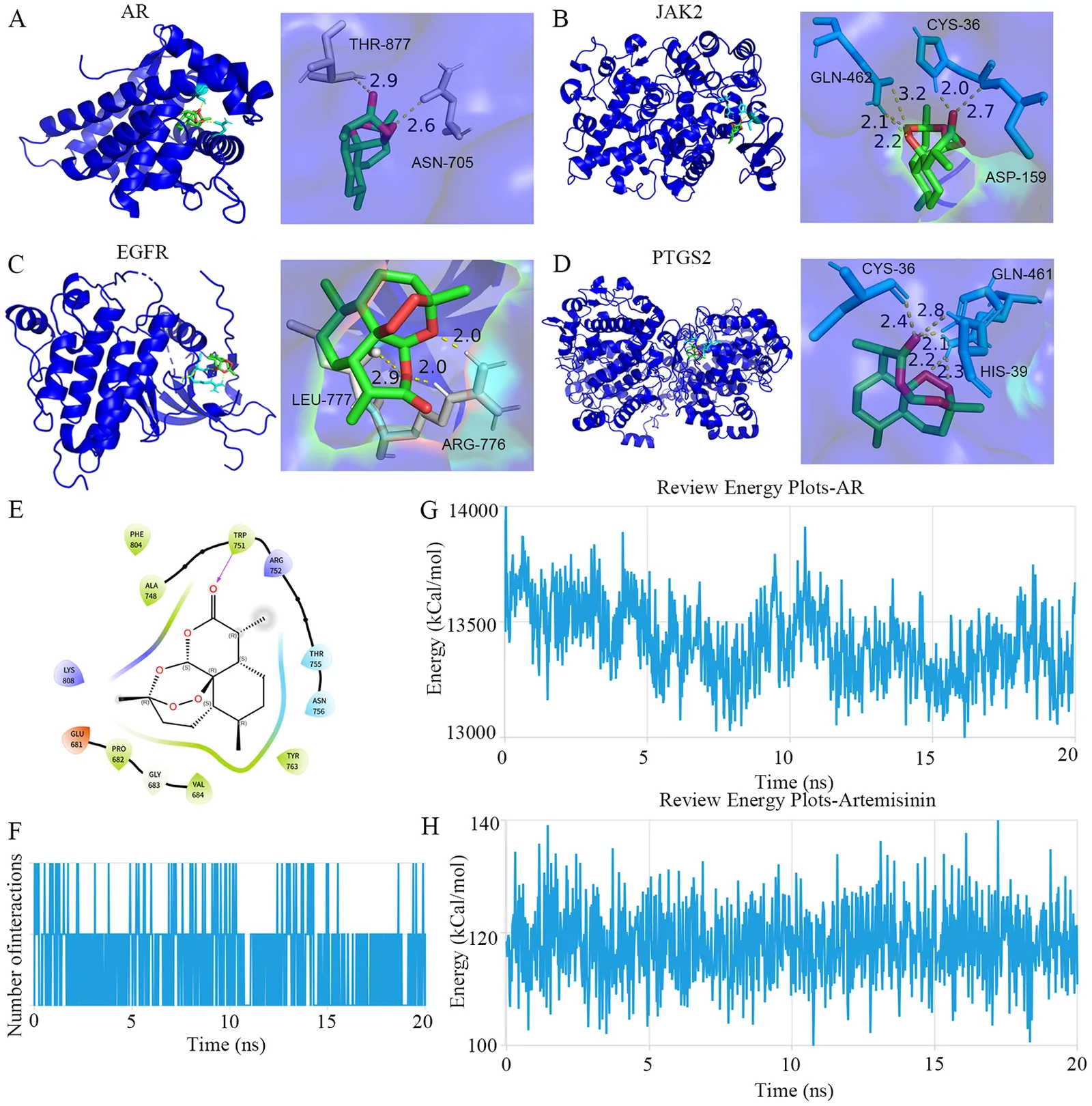
Molecular docking and molecular dynamics analysis of artemisinin with candidate hub targets. **(A–D)** Predicted binding modes of artemisinin with AR **(A)**, EGFR **(B)**, JAK2 **(C)**, and PTGS2 **(D)** obtained by molecular docking. **(E)** Detailed interaction pattern of the artemisinin–AR complex. **(F)** Protein–ligand contact analysis during molecular dynamics simulation. **(G)** Energy fluctuation profile of the AR–artemisinin complex during the simulation. **(H)** Energy fluctuation profile of artemisinin during the simulation period.

Molecular docking simulations were performed between artemisinin and four key targets, including AR (PDB ID: 2piv), JAK2 (PDB ID: 3tjd), EGFR (PDB ID: 5fed) and PTGS2 (PDB ID: 5ikq). Molecular docking analysis predicted favorable binding affinities between artemisinin and the network-prioritized candidate targets (**Table 1**). Molecular dynamics simulations suggested relatively stable interaction of the artemisinin-AR complex (binding energy: -8.65 kcal/mol; **Figure 5A**; **Table 1**), while compact binding conformations were observed within the hydrophobic clefts of EGFR (**Figure 5B**) and JAK2 (**Figure 5C**), demonstrating favorable ligand-receptor complementarity. Artemisinin with amino acid residues, including Cys36, Asp159, and GLN462, formed five hydrogen bonds, allowing artemisinin and JAK2 to constitute a stable complex (**Figure 5B**). Artemisinin was predicted to bind EGFR through interactions with Arg776 and Leu777, forming three hydrogen bonds (**Figure 5C**). Similarly, artemisinin was predicted to interact with PTGS2 and form five hydrogen bonds involving Cys36, His39, and Gln461 (**Figure 5D**). Considering that artemisinin exhibited the lowest binding energy with AR, we further analyzed their interaction. **Figure 5E** shows that artemisinin fits well into the binding pocket of the AR and forms multiple interactions with the surrounding key amino acid residues, suggesting a favorable binding mode. The molecular dynamics results further indicated that the AR–artemisinin complex maintained a certain number of interactions throughout the 20 ns simulation (**Figure 5F**), supporting the persistence of binding. In addition, the energy profiles of both the AR complex and the artemisinin complex exhibited only minor fluctuations without obvious abnormal drift during the simulation period (**Figure 5G, H**; **Supplementary Video 1**), indicating that both complexes remained relatively stable. Overall, these in silico results suggest a potential interaction between artemisinin and selected candidate hub targets, which requires further experimental validation.

**Table 1:** Molecular docking binding energy results (kcal/mol).

Ligand	AR	JAK2	EGFR	PTGS2
Artemisinin	–8.65	–8.61	–7.48	–8.48

### 3.6 Artemisinin suppresses IL-17-induced inflammatory activation and PI3K/AKT signaling in MH7A cells

**Figure 6:**
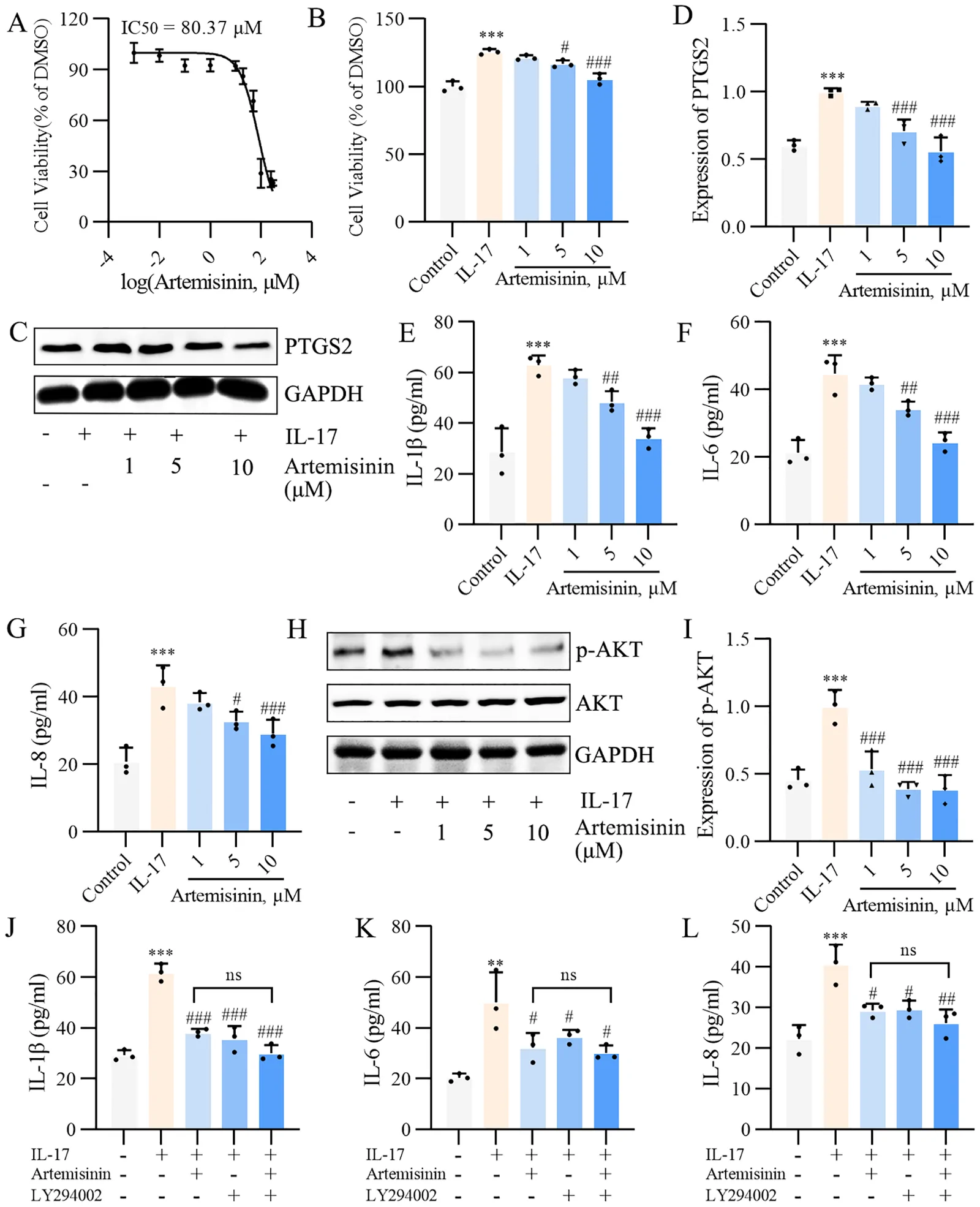
Artemisinin inhibits IL-17-induced inflammatory response. **(A)** MH7A cell viability was evaluated by CCK-8 after treatment with artemisinin at varying concentrations for 24 h. **(B)** Viability of MH7A cells following 2-hour artemisinin pre-treatment and 24 h IL-17 stimulation. **(C, D)** Representative western blots showing the effect of artemisinin on IL-17-induced PTGS2 expression **(C)**, which was quantified using ImageJ software **(D)**. **(E-G)** After the treatment with various concentrations of artemisinin for 24 h in MH7A, IL-1β **(E)**, IL-6 **(F)** and IL-8 **(G)** were examined by ELISA, respectively. **(H)** Representative western blots showing AKT phosphorylation status in MH7A cells after co-incubation with IL-17 and artemisinin for 2 hours. **(I)** Quantitative analysis of AKT phosphorylation levels shown in **(H)** using ImageJ software. **(J–L)** Effects of artemisinin (10 μM), LY294002 (10 μM), or their combination on IL-17-induced secretion of IL-1β **(J)**, IL-6 **(K)**, and IL-8 **(L)** in MH7A cells. Data are presented as mean ± SD, n = 3. Statistical significance was determined by one-way ANOVA followed by Tukey’s multiple comparisons test. ^**^p < 0.01, ^***^p < 0.001 vs. control group; ^#^p < 0.05, ^##^p < 0.01, ^###^p < 0.001 vs. IL-17 group.

To confirm the mechanisms of artemisinin in RA suggested by bioinformatics, validation experiments were carried out. Viability testing showed that significant inhibition of MH7A cells occurred only when artemisinin was applied at 50 μM, supporting its cellular tolerance (**Figure 6A**). KEGG pathway analysis revealed artemisinin-mediated modulation of the IL-17 signaling axis, prompting mechanistic investigation into its regulatory effects on this inflammatory cascade. **Figure 6B** indicated that artemisinin significantly inhibited IL-17-induced cell proliferation at 5 μM. Additionally, the expression of IL-17-induced PTGS2 was also inhibited by artemisinin (**Figures 6C, D**). Furthermore, the production of inflammatory cytokines induced by IL-17 was reversed by artemisinin, including IL-1β, IL-6, and IL-8 (**Figures 6E–G**). Given that the PI3K/AKT pathway acts as a major downstream mediator of IL-17 and contributes to critical pathogenic mechanisms in RA, we assessed how artemisinin influences this signaling cascade. Immunoblotting revealed IL-17-induced AKT phosphorylation, which was dose-dependently suppressed by artemisinin (**Figures 6H, I**). To further evaluate the role of PI3K/AKT signaling in the anti-inflammatory activity of artemisinin, MH7A cells were treated with the PI3K/AKT pathway inhibitor LY294002 under IL-17 stimulation. IL-17 markedly increased the secretion of IL-1β, IL-6, and IL-8, whereas artemisinin significantly reduced the levels of these cytokines. Similarly, pharmacological inhibition of PI3K/AKT signaling decreased IL-17-induced cytokine production. The combined treatment with artemisinin and LY294002 did not produce a markedly stronger inhibitory effect than pathway inhibition alone (**Figures 6J–L**), suggesting that artemisinin may exert its anti-inflammatory effect, at least partly, through suppression of the PI3K/AKT pathway. These findings provide additional pharmacological evidence supporting the involvement of PI3K/AKT signaling in the regulation of IL-17-induced inflammatory activation by artemisinin.

### 3.7 Artemisinin inhibits CIA

**Figure 7:**
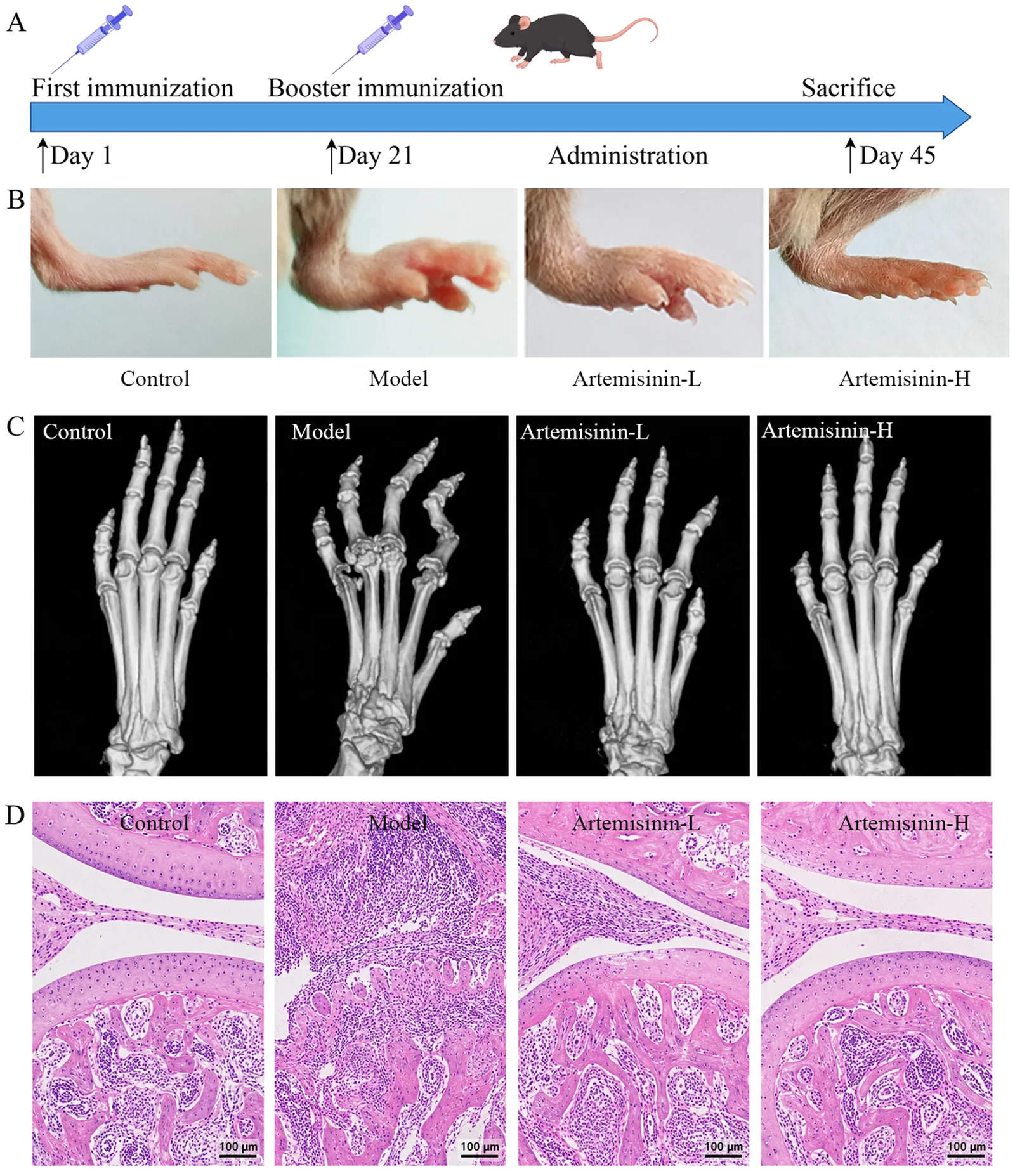
Artemisinin ameliorates CIA progression in mice. **(A)** Schematic representation of the experimental timeline for CIA induction and drug administration. **(B)** Representative photographs of hind paws from each group at day 45. **(C)** Representative micro-CT images of tibiotarsal joints from the Control, Model (CIA), CIA + Artemisinin-L, and CIA + Artemisinin-H groups. **(D)** Representative HE-stained sections of tibiotarsal joints from the Control, Model (CIA), CIA + Artemisinin-L, and CIA + Artemisinin-H groups (Scale bar = 100 μm). Artemisinin-L = 15 mg/kg, Artemisinin-H = 30 mg/kg, n = 6 per group.

CIA model was used to validate the therapeutic effects of artemisinin in rheumatoid arthritis. Co-administration of artemisinin commenced concurrently with secondary intradermal immunization using incomplete Freund’s adjuvant (**Figure 7A**). Bovine collagen type II provoked pronounced hind paw erythema and edema in mice, which were markedly attenuated following artemisinin intervention (**Figure 7B**). Micro-CT imaging revealed severe bone erosion, joint-space narrowing, and structural deformation in the joints of CIA model mice. Artemisinin treatment attenuated these bone-destructive changes in a dose-dependent manner (**Figure 7C**). HE staining showed that CIA mice exhibited obvious synovial hyperplasia, inflammatory cell infiltration and cartilage erosion compared with control mice. Artemisinin attenuated these pathological changes, especially at the high dose (**Figure 7D**). These results suggest that artemisinin protects against CIA-induced joint inflammation and tissue destruction.

**Figure 8:**
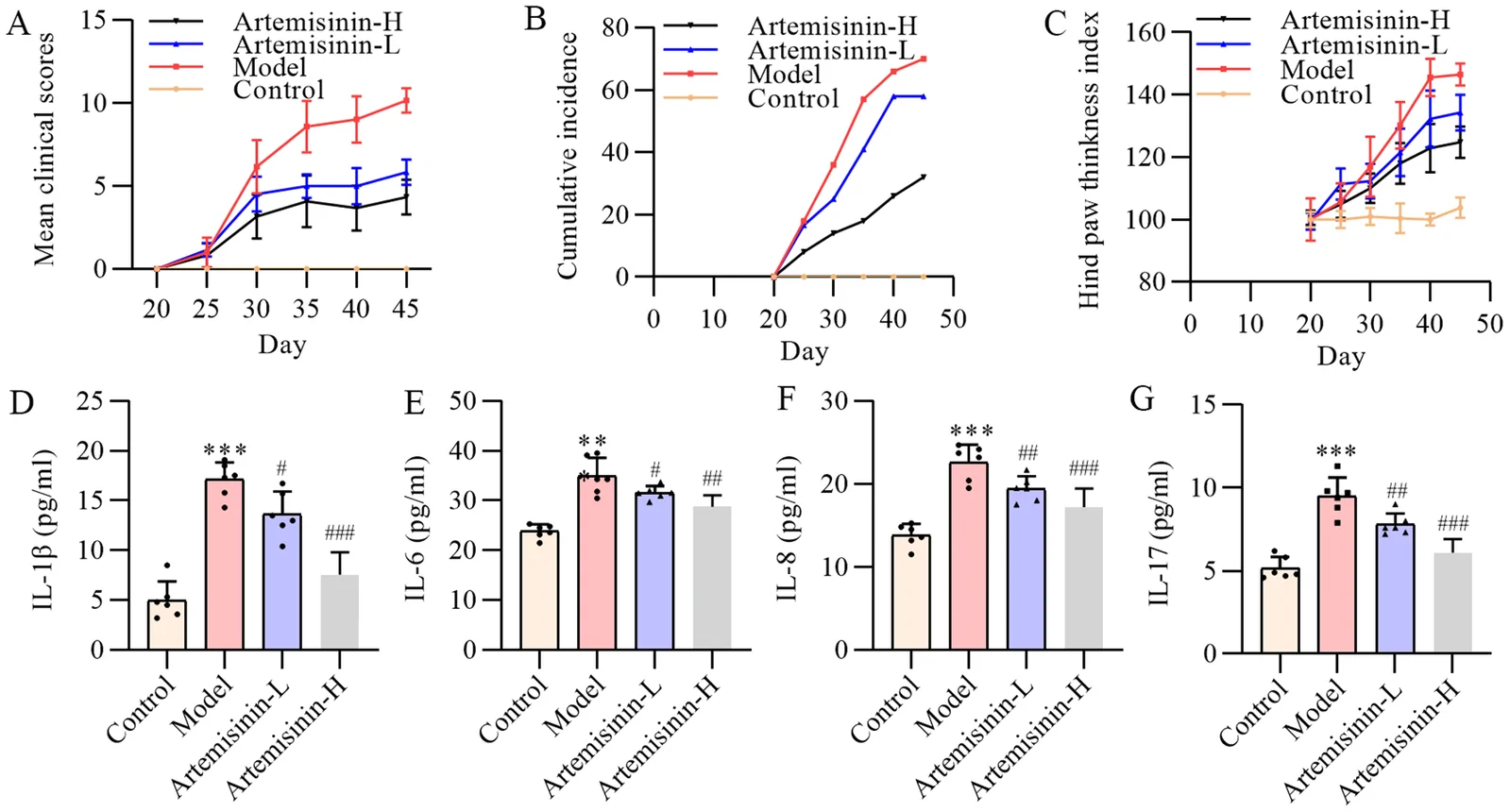
Artemisinin attenuates arthritis progression and systemic inflammatory responses in CIA mice. **(A)** Mean clinical arthritis scores of mouse hind paws. **(B)** Cumulative incidence rate of arthritis in the CIA model. **(C)** Changes in hind paw thickness over the course of the experiment. **(D–G)** Serum levels of IL-1β **(D)**, IL-6 **(E)**, IL-8 **(F)**, and IL-17 **(G)** after treatment. Data is presented as mean ± SD; n = 6 per group. Statistical significance was determined by one-way ANOVA followed by Tukey’s multiple comparisons test. ^**^p < 0.01, ^***^p < 0.001 vs. control group; ^#^*p* < 0.05, ^##^*p* < 0.01, ^###^*p* < 0.001 vs. CIA group. Artemisinin-L = 15 mg/kg, Artemisinin-H = 30 mg/kg.

Quantitative clinical assessment revealed collagen/adjuvant-induced elevation of mean arthritis scores, an effect significantly suppressed by artemisinin treatment (**Figure 8A**). Similarly, artemisinin administration reduced cumulative arthritis incidence and decreased paw thickness indices relative to vehicle-treated CIA controls (**Figures 8B, C**). Molecular profiling of serum inflammatory mediators demonstrated artemisinin’s capacity to reverse CIA-elevated levels of IL-1β, IL-6, and IL-8 (**Figures 8D–F**). Mechanistic investigation further confirmed dose-dependent suppression of IL-17 production (**Figure 8G**), substantiating pathway modulation observed in prior KEGG analyses.

## 4 Discussion

Artemisinin, a prototypical small-molecule therapeutic isolated from the traditional Chinese medicinal herb *Artemisia annua*, is a sesquiterpene lactone featuring a crucial peroxide bridge that confers its core antimalarial activity (7, 20–24). Although clinical trials of artemisinin itself in RA are lacking, *A. annua extract* has shown potential benefits in RA and osteoarthritis (25–27). Preclinical evidence further indicates artemisinin’s therapeutic potential for RA (12), with formulation engineering substantially augmenting its anti-arthritic efficacy (28). Consistently, artemisinin analogs, including artesunate (29) and dihydroartemisinin (15) are under intensive investigation for RA treatment (12, 30, 31). By integrating artemisinin target prediction with RA synovial transcriptomic profiles, we identified 73 overlapping candidate targets and prioritized AR, EGFR, JAK2, and PTGS2 as hub targets. However, these docking results should be interpreted cautiously because systematic comparison with known inhibitors or reference ligands was not performed. Experimental validation confirmed that artemisinin inhibited IL-17-induced synovial fibroblast proliferation, supporting its potential therapeutic mechanism against RA. Although MH7A cells provided a stable RA-FLS-like model, validation in primary RA-FLSs remains necessary. Although we added a low-dose artemisinin group, micro-CT analysis, and histopathological assessment to strengthen the *in vivo* evidence, this study still lacked pharmacokinetic analysis, systematic safety evaluation, and comparison with standard DMARDs or biologics. Future studies should address these issues to better clarify the therapeutic window, safety profile, and translational potential of artemisinin in RA.

Artemisinin exerts its antimalarial activity primarily by reacting with free iron or heme in the *Plasmodium* digestive vacuole, inducing extensive parasitic protein damage (20). Additional targets include the Kelch13 protein (32), TCTP (20), and PfATP6 (33), demonstrating its polypharmacological nature. Consistently, multiple targets have been identified across diverse disease contexts: in inflammatory diseases, artemisinin targets myeloid differentiation factor 2 and heat shock protein 90 to exert anti-inflammatory effects (10, 34, 35). In neurodegenerative disorders, it inhibited neuronal ferroptosis in Alzheimer’s models through KEAP1 targeting (36). In oncology, the compound suppressed NRAS palmitoylation by inhibiting ZDHHC6 acyltransferase (37), and notably in RA, PKM2 emerged as a potential therapeutic target, though direct mechanistic evidence remains to be fully elucidated. Our molecular docking predicted high-affinity interactions between artemisinin and RA-relevant proteins, including AR (38), JAK2 (39), EGFR (40–42) and PTGS2 (43). These findings support the polypharmacological potential of artemisinin and suggest that AR, EGFR, JAK2, and PTGS2 may represent computationally prioritized candidate targets for further mechanistic investigation in RA. Nevertheless, AR, EGFR, JAK2, and PTGS2 remain computationally predicted candidate targets rather than experimentally confirmed direct targets. Although LY294002 supported the involvement of PI3K/AKT signaling, further target-specific and rescue experiments are needed to clarify their causal roles in RA-FLSs.

IL-17 is a highly pleiotropic pro-inflammatory cytokine, primarily secreted by Th17 cells. It directly participates in local joint inflammation and systemic pathological processes, thereby exacerbating RA progression (44). In preclinical models, IL-17-targeting antibodies significantly ameliorate arthritis symptoms and neutralize elevated IL-17 levels in patient serum (45). Furthermore, IL-1β can indirectly promote IL-17-mediated bone erosion by inducing Treg cells to acquire osteoclastogenic activity (46). Our experimental validation also confirms the critical role of IL-17, demonstrating for the first time that artemisinin suppresses its mediated expression and activation in synovial fibroblasts. This mechanism was also substantiated in the mechanisms of action of other drugs (47, 48). The EGFR-regulated downstream PI3K/AKT pathway is concurrently activated by IL-17; therefore, we assessed AKT protein phosphorylation. Western blotting results showed that artemisinin attenuated IL-17-induced AKT phosphorylation, providing experimental support for the involvement of PI3K/AKT signaling suggested by enrichment analysis. The LY294002 experiment further showed that pharmacological inhibition of PI3K/AKT mimicked the inhibitory effect of artemisinin on IL-17-induced cytokine production, supporting the involvement of this pathway in artemisinin-mediated suppression of FLS inflammatory activation. Notably, previous studies have reported that artemisinin derivatives regulate inflammatory responses, Th17/Treg balance, and PI3K/AKT-related signaling (13, 30, 49–52). In contrast, our study focused on artemisinin itself and linked human RA synovial transcriptomic analysis, candidate-target prediction, IL-17-stimulated MH7A validation, and CIA mouse phenotypes. These findings provide a more integrated framework for understanding artemisinin-mediated anti-inflammatory activity in RA.

## 5 Conclusions

In summary, this study suggests that artemisinin exerts anti-RA effects partly by suppressing IL-17-induced inflammatory activation and PI3K/AKT signaling. Integrated network pharmacology and molecular docking prioritized AR, EGFR, JAK2, and PTGS2 as candidate hub targets, while cellular and CIA mouse experiments supported the anti-inflammatory and joint-protective effects of artemisinin. Further studies are required to confirm direct target engagement and clinical relevance in primary RA-FLSs and comparative therapeutic models.

## Data Availability

The original contributions presented in the study are included in the article/**Supplementary Material**. Further inquiries can be directed to the corresponding authors.
