# Supplementary material for: Artemisinin ameliorates rheumatoid arthritis through modulation of the IL-17/PI3K/AKT signaling pathway: integrated network pharmacology, bioinformatics, and experimental validation
Source: Front Immunol. 2026 Aug 13;17:1862104. doi: 10.3389/fimmu.2026.1862104 (PMC13518343; doi:10.3389/fimmu.2026.1862104)
Supplement: Supplementary file 1 [file DataSheet1.pdf]

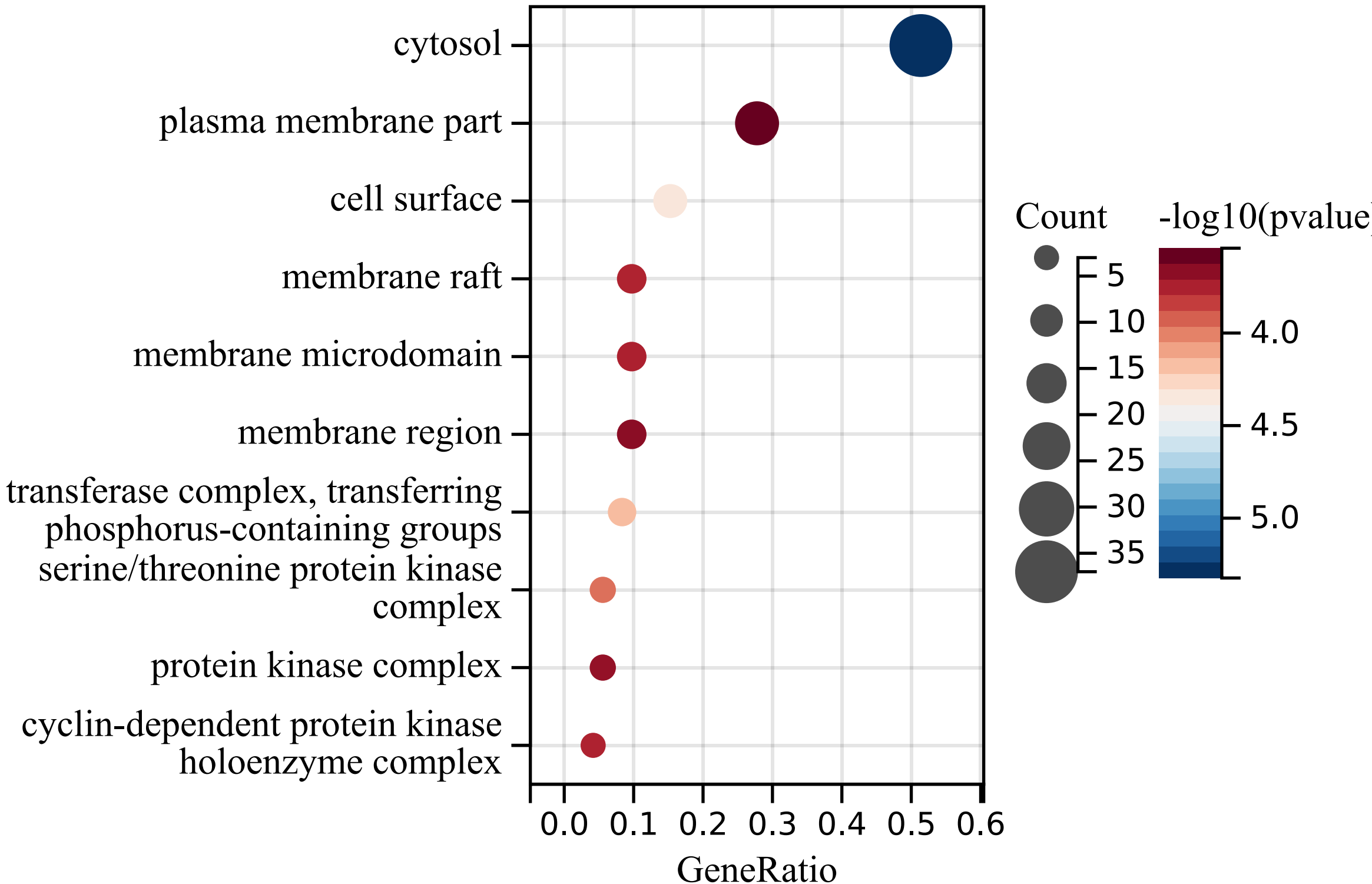
cytosol
plasma membrane part
cell surface
membrane raft
membrane microdomain
membrane region
transferase complex, transferring phosphorus-containing groups
serine/threonine protein kinase complex
protein kinase complex
cyclin-dependent protein kinase holoenzyme complex
0.0
0.1
0.2
0.3
0.4
0.5
0.6
GeneRatio
Count
5
10
15
20
25
30
35
-log10(pvalue)
4.0
4.5
5.0
